# Salt reduction behavior of adults in Anhui province in 2019: a cross-sectional survey of 3,378 participants

**DOI:** 10.3389/fpubh.2023.1242969

**Published:** 2023-10-16

**Authors:** Xiu-Ya Xing, Yan Zhao, Napoleon Bellua Sam, Jing-Qiao Xu, Ye-Ji Chen, Wei Xu, Hua-Dong Wang, Zhi-Rong Liu, Hai-Feng Pan

**Affiliations:** ^1^Department of Chronic Non-Communicable Disease Prevention and Control, Anhui Provincial Center for Disease Control and Prevention, Hefei, China; ^2^Department of Epidemiology and Biostatistics, School of Public Health, Anhui Medical University, Hefei, Anhui, China; ^3^Institute of Kidney Disease, Inflammation and Immunity-Mediated Diseases, The Second Hospital of Anhui Medical University, Hefei, Anhui, China; ^4^Department of Medical Research and Innovation, School of Medicine, University for Development Studies, Tamale, Ghana

**Keywords:** salt intake reduction, hypertension, behavior, salt intake, diet

## Abstract

**Objective:**

A high-sodium diet is an important risk factor for hypertension in the Chinese population, which can increase the risk of cardiovascular and cerebrovascular diseases. Although a large number of related studies have been carried out in Anhui province, clear, effective salt reduction interventions and policies that can be widely promoted have not yet been formed. This study sought to understand the prevalence and precise measures of salt reduction behavior, the variables affecting salt reduction behavior, and the reasons why salt reduction behavior was not practiced in Anhui Province, China.

**Methods:**

The total number of participants in the study was 3,378. Using a multi-stage stratified cluster random sampling method, residents between the ages of 18 and 69 years in 10 counties and districts were selected from March to October 2019. A survey questionnaire and physical measurements were given to each participant. The influencing factors of residents' salt reduction behavior were examined using a multi-factor unconditional logistic regression analysis. The chi-squared (χ^2^) test was used to analyze the implementation of salt reduction behaviors among different age groups and gender, the factors influencing the implementation of salt reduction measures, and the reasons for not implementing salt reduction measures.

**Results:**

A history of hypertension was associated with salt reduction strategies (*P* = 0.014). Patients with hypertension were more likely to adopt salt reduction behaviors than those without hypertension (*OR* = 1.218, *P* = 0.040). The influence of eating out on the adoption of salt-reduction measures varied by age group (χ^2^ = 50.463, *P* < 0.001) and gender (χ^2^ = 81.348, *P* < 0.001).

**Conclusion:**

In summary, residents of the Anhui Province are not very knowledgeable about salt reduction. Age, gender, education level, hypertension, and marital status are the main determinants. Our findings have significant implications for policymakers who want to devise salt reduction strategies.

## 1. Introduction

In 2012, the average daily salt intake for individuals over the age of 18 years in China, one of the countries with the greatest salt consumption, was 10.5 g, which was approximately double the amount recommended by the Chinese Dietary Guidelines (6 g) or the WHO (5 grams) (1). Excessive salt consumption might increase the risk of chronic diseases such as hypertension and aggravate morbidity and mortality. High sodium intake, one of the top three dietary risk factors, contributed to 3 million deaths and 70 million years lost to disability-adjusted life globally in 2017 alone (2). The strong association between salt intake and hypertension has been confirmed by many studies, with results showing a positive relationship between salt intake and blood pressure (3–5). Some other studies have also shown associations between high salt intake and obesity and stroke (6, 7).

Reducing salt consumption is among the most affordable, practical, and accessible ways to prevent cardiovascular disease (CVD), and more than 70 countries have already launched various salt reduction initiatives (8). In 2013, the WHO recommended that all member states aim to reduce population salt intake by 30% by 2025 (9). The UK was among the first nations to undertake a salt reduction strategy to lower the risk of CVD. It started off well, but recently, it has halted without accomplishing its national and global goals (10). Despite the release of several guidelines promoting salt reduction, the majority of the Chinese population still consumes high amounts of salt. However, there is limited literature on the population's current salt reduction strategies and the variables affecting salt consumption.

To understand the current state of residents' salt reduction behaviors, analyze the factors affecting the adoption of salt reduction measures, and determine the reasons for not adopting salt reduction measures, this study evaluated salt reduction behaviors of residents in 10 counties and districts in the Anhui Province from March to October 2019. As a result of the impending assessment of the program in Anhui Province for the prevention and control of hypertension and salt reduction, some recommendations would be made.

## 2. Material and methods

### 2.1. Study population

**Figure 1:**
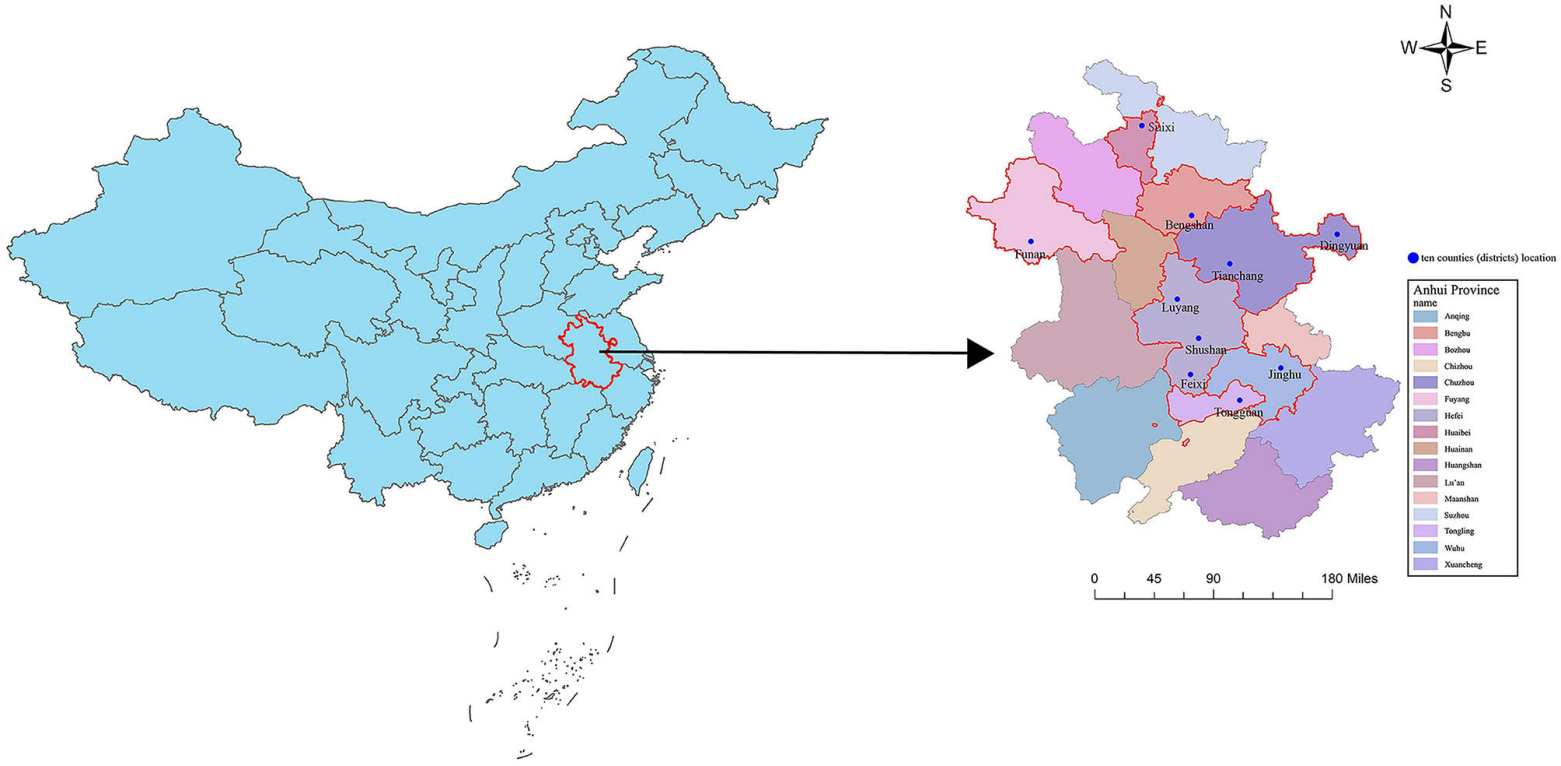
Map of study sites in Anhui province of China.

Participants for this study were selected from 10 counties and districts of Anhui province between March and October 2019 (Figure 1). The residents aged 18 to 69 years old were selected as the survey population, and the permanent residents lived in the area for more than 6 months. Those with serious physical diseases who were unable to participate in the survey were excluded. The study protocol was approved by the medical ethics review committee of the Anhui Provincial Center for Disease Control and Prevention. Informed consent was obtained from all study subjects.

### 2.2. Sampling method

A multi-stage cluster sampling procedure was adopted for the survey. Ten counties (districts) were selected according to geographical location and local health work status for the first stage of the survey. In the second stage, the Probability Proportionate to Size Sampling (PPS) method was proportional to the population size, which was used to randomly select three townships (streets) from each county (district). In the third stage, the cluster sampling method of PPS was used to randomly select five villages (neighborhood committees) from each township (street). A simple random sampling method was adopted to randomly select 25 people from each village (neighborhood committee) to ensure the representativeness of the participants.

### 2.3. Survey contents

An electronic questionnaire was used to carry out the survey. This survey mainly included the following contents: (1) fundamental demographic information such as gender, age, education level, etc.; (2) salt reduction-related behaviors, including whether salt reduction measures had been taken and the specific measures taken, factors influencing the adoption of salt reduction measures, and reasons for not taking salt reduction measures; (3) measurements of the body, such as height, weight, waist circumference (WC), blood pressure (BP), etc. A 1.5-m tape measure was used to measure WC, which was accurate to 0.1 cm. The height was measured using a Seca 213 height-measuring instrument with a 1-mm graduation length. The Seca 813 mobile electronic fat scales were used to measure weight while wearing light clothing and to the nearest 0.1 kg. BP measurements were estimated to be 1 mmHg (Omron HBP-1100-E; OMRON Healthcare, Hoofddorp, the Netherlands). After a 3-min rest, BP measurements were repeated three times, and the average systolic and diastolic BP of the three readings were recorded on the questionnaire. The mean BP values of the three repeated measurements were included in the final analysis. All data were measured by the trained investigators.

### 2.4. Definition of indicators

Overweight and adiposity were defined by body mass index (BMI) (11). Participants were divided into four categories based on their weight: underweight (<18.5 kg/m^2^), normal (18.5– <24.0 kg/m^2^), overweight (24.0– <28.0 kg/m^2^), and obese (≥28.0 kg/m^2^) (12) Men with WC ≥90 cm and women with WC ≥ 85 cm were considered to have central obesity (13) Hypertension was defined as one of the following two conditions: (1) systolic BP (SBP) ≥140 mmHg and/or diastolic BP (DBP) ≥90 mmHg; (2) taking antihypertensive medicine within 2 weeks at the time of the investigation (14).

### 2.5. Quality control

The questions in the questionnaire were easy to understand, and the use of professional words was avoided. Experienced personnel were selected to participate in the survey, and relevant training was conducted before the formal survey to ensure the investigators were clear about the purpose, content, and significance of the survey. During the survey, when the participants did not understand the areas, the investigators should give timely and patient explanations. Furthermore, the questionnaires were filled out by the investigators. After the survey, the survey forms were reviewed, sorted out, and archived by the provincial data management team.

### 2.6. Statistical analysis

Microsoft Office Excel 2007 was used to establish the database, and the original data were entered using unified standards and procedures. Count data were described by frequency and constituent ratio, and the chi-squared (χ^2^) test was used to compare groups. A univariate logistic regression analysis was performed with gender, age, education level, occupation, marital status, living status, BMI, central obesity, and family history of hypertension as independent variables. The presence or absence of salt reduction measures was used as the dependent variable. The statistically significant factors in the univariate analysis were used as independent variables to conduct a multivariate unconditional logistic regression analysis (forward: LR method) to explore the influencing factors of residents' salt reduction measures. All analyses above were conducted using R software (version: 3.4.0), with a two-tailed *P* < 0.05 indicating statistical significance.

## 3. Results

### 3.1. Descriptive statistics

The characteristics of the participants have been summarized in Table 1, with an almost equal distribution of male and female participants of 47.31 and 52.69%, respectively. A total of 3,378 valid questionnaires were retrieved for the study. The majority of participants were aged 30–44 years (40.79%) and 45–59 years (27.56%). People at the junior high school level made up the largest proportion (29.25%). Farmers and other workers made up the majority of occupations (31.53%). The majority of people (86.62%) were married. Urban and rural areas had respective populations of 46.74 and 53.26%. Additionally, individuals who had hypertension, central adiposity, and obesity made up 21.40, 39.08, and 13.94% of the population, respectively.

**Table 1:** Univariate analysis of salt reduction behavior holding rate among people with different characteristics.

**Variables**	**Total (*n =* 3,378)**	**Not holding salt reduction behavior (*n =* 1,349)**	**Holding salt reduction behavior (*n =* 2,029)**	***χ^2^***	***P*-value**
**Gender (%)**				58.650	< 0.001
Men	1,598 (47.31)	747 (55.37)	851 (41.94)		
Women	1,780 (52.69)	602 (44.63)	1,178 (58.06)		
**Age [years (%)]**				30.357	< 0.001
18~29	597 (17.68)	296 (21.94)	301 (14.83)		
30~44	1,378 (40.79)	541 (40.10)	837 (41.25)		
45~59	931 (27.56)	339 (25.13)	592 (29.18)		
60~69	472 (13.97)	173 (12.83)	299 (14.74)		
**Education level (%)**				10.674	0.030
Below primary school	597 (17.67)	230 (17.05)	367 (18.09)		
Primary school	412 (12.20)	180 (13.34)	232 (11.44)		
Middle school	988 (29.25)	405 (30.02)	583 (28.73)		
High school	598 (17.70)	254 (18.83)	344 (16.95)		
College or above	783 (23.18)	280 (20.76)	503 (24.79)		
**Career (%)**				26.956	< 0.001
Farmer/Workers	1,065 (31.53)	451 (33.43)	614 (30.26)		
Business/Service industry	539 (15.96)	201 (14.90)	338 (16.66)		
Administrative staff	324 (9.59)	122 (9.04)	202 (9.96)		
Professional technicians	308 (9.12)	124 (9.20)	184 (9.07)		
Students	110 (3.25)	64 (4.74)	46 (2.27)		
Retired	223 (6.60)	97 (7.19)	126 (6.20)		
Other	809 (23.95)	290 (21.50)	519 (25.58)		
**Marital status (%)**				20.144	< 0.001
Unpartnered	452 (13.38)	224 (16.60)	228 (11.24)		
Partnered	2,926 (86.62)	1,125 (83.40)	1,801 (88.76)		
**Region (%)**				4.942	0.026
Rural	1,799 (53.26)	750 (55.60)	1,049 (51.70)		
Urban	1,579 (46.74)	599 (44.40)	980 (48.30)		
**BMI (kg/m** ^2^ **, %)**				5.154	0.161
Underweight	128 (3.79)	62 (4.60)	66 (3.25)		
Normal	1,554 (46.00)	610 (45.22)	944 (46.53)		
Overweight	1,225 (36.27)	498 (36.91)	727 (35.83)		
Obesity	471 (13.94)	179 (13.27)	292 (14.39)		
**Central obesity (%)**				1.654	0.198
Yes	1,320 (39.08)	545 (40.40)	775 (38.20)		
No	2,058 (60.92)	804 (59.60)	1,254 (61.80)		
**Hypertension (%)**				6.055	0.014
No	2,655 (78.60)	1,089 (80.73)	1,566 (77.18)		
Yes	723 (21.40)	260 (19.27)	463 (22.82)		

### 3.2. Univariate analysis of residents' salt reduction behavior

According to Table 1, residents aged 18–69 years had a salt reduction behavior rate of 60.07% (2,029/3,378). Different salt reduction rates by gender, age, occupation, and marital status were observed (all *P* < 0.001). There were differences in salt reduction rates according to educational level (*P* = 0.030). Furthermore, there were regional differences in the rates of salt reduction behavior (*P* = 0.026). Hypertension was associated with a difference in the use of salt reduction measures (*P* = 0.014), and people with high BP were more likely to take salt reduction measures. There were no significant differences in salt reduction behavior rates among people with different BMI levels or those with and without central obesity.

### 3.3. Multi-factor analysis of the adoption of salt reduction behaviors

According to the *P* < 0.05 criteria, a multifactorial logistic regression was built using seven variables. According to Table 2, the salt reduction behavior rate was higher in women than in men (*OR* = 1.816, *P* < 0.001). There was an increasing trend for salt reduction behavior rate with the population aging (*P*_*trend*_ < 0.001). Those with junior high school education or below were 1.306 times more likely to take salt reduction measures than those with primary school education or below (*P* = 0.026), and those with a college education or above were 1.773 times more likely to take measures to reduce salt than those with primary school education or below (*P* < 0.001). Hypertensive patients were 1.218 times more likely to adopt salt reduction behavior than non-hypertensive patients (*P* = 0.040). Married people were 1.277 times more likely to take measures to reduce salt than unmarried people (*P* = 0.036).

**Table 2:** Multivariate logistic regression analysis of adult residents taking the initiative to take salt reduction measures in Anhui Province.

**Variables**	**Beta**	**S.E**.	**Wald *χ^2^***	***OR* (95% CI)**	***P*-value**
**Gender**					
Men				1.000 (reference)	
Women	0.596	0.074	64.819	1.816 (1.570, 2.099)	< 0.001
**Age (years)** ^a^					
18~29			23.299	1.000 (reference)	< 0.001
30~44	0.360	0.112	10.434	1.434 (1.152, 1.784)	0.001
45~59	0.567	0.127	20.026	1.762 (1.375, 2.259)	< 0.001
60~69	0.644	0.154	17.578	1.904 (1.409, 2.573)	< 0.001
**Education level**					
Below primary school			26.637	1.000 (reference)	< 0.001
Primary school	−0.002	0.137	0.000	0.998 (0.763, 1.306)	0.989
Junior high school	0.267	0.120	4.939	1.306 (1.032, 1.653)	0.026
High school	0.249	0.131	3.616	1.283 (0.992, 1.659)	0.057
College or above	0.572	0.131	19.179	1.773 (1.372, 2.290)	< 0.001
**Marital status**					
Unpartnered				1.000 (reference)	
Partnered	0.245	0.116	4.410	1.277 (1.016, 1.604)	0.036
**Hypertension**					
No				1.000 (reference)	
Yes	0.197	0.096	4.197	1.218 (1.009, 1.470)	0.040

^a^P for trend = 0.000.

### 3.4. Analysis of specific measures to reduce salt

As shown in Table 3, “use less salt or high-salt condiments when cooking at home” and “add salt at a later time when cooking” were the most and least effective salt reduction strategies, respectively (90.98 vs. 31.35%). Compared to men, women were more likely to forego or limit eating out (*P* < 0.001). There was a significant difference in “avoid or eat fewer pickles and other pickled products” among different age groups (*P* < 0.001). Moreover, significant differences in “avoid or limit processed foods” and “use scallions, ginger, and garlic to enhance flavor and reduce salt usage” were observed among various age groups (all *P* < 0.050).

**Table 3:** Comparison of specific salt reduction measures by gender and age groups.

**Salt reduction measures adopt(multiple choices)**	**Total (*n =* 2,029)**	**Gender**				**Age (years)**					
		**Men (*****n** =* **851)**	**Women (*****n** =* **1,178)**	χ^2^	***P-*** **value**	**18**~**29 (*****n** =* **301)**	**30**~**44 (*****n** =* **837)**	**45**~**59 (*****n** =* **592)**	**60**~**69 (*****n** =* **299)**	χ^2^	***P-*** **value**
Use less salt or high-salt condiments when cooking at home (%)	1,846 (90.98)	765 (89.89)	1,081 (91.77)	2.109	0.146	264 (87.71)	768 (91.76)	539 (91.05)	275 (91.97)	4.905	0.179
Avoid or limit processed foods (%)	1,200 (59.14)	500 (58.75)	700 (59.42)	0.091	0.762	169 (56.15)	522 (62.37)	348 (58.78)	161 (53.84)	8.219	0.042
Avoid or eat fewer pickles and other pickled products (%)	1,173 (57.81)	490 (57.58)	683 (57.98)	0.032	0.857	196 (65.12)	506 (60.45)	319 (53.89)	152 (50.84)	18.688	< 0.001
Avoid or reduce eating out (%)	978 (48.20)	368 (43.24)	610 (51.78)	14.430	< 0.001	134 (44.52)	420 (50.18)	283 (47.80)	141 (47.16)	3.115	0.374
Use scallions, ginger, and garlic to enhance flavor and reduce salt usage (%)	790 (38.94)	318 (37.37)	472 (40.07)	1.515	0.218	106 (35.22)	357 (42.65)	226 (38.18)	101 (33.80)	10.102	0.018
Add salt at a later time when cooking (%)	636 (31.35)	258 (30.32)	378 (32.09)	0.720	0.396	97 (32.23)	273 (32.62)	186 (31.42)	80 (26.76)	3.665	0.300

### 3.5. Analysis of the factors influencing the adoption of salt reduction measures

The factors listed in Table 4 with the highest and lowest percentages of choices were “not knowing the salt content of the commonly eaten foods” and “too few low-salt foods in supermarkets” (47.46 vs. 24.46%). Men had a significant advantage over women when it came to “eating out frequently” when gender was taken into consideration (*P* < 0.001). A subgroup analysis by age revealed that “can't understand the meaning of salt content on food nutrition labels,” “too few low-salt dishes in restaurants,” “eat out frequently,” and “too few low-salt foods in supermarkets” differed significantly among different age groups (all *P* < 0.001).

**Table 4:** Comparison of the factors influencing the adoption of salt reduction measures among different gender and age groups.

**Factors influencing the adoption of salt reduction measures (multiple choices)**	**Total (*n =* 2,029)**	**Gender**				**Age (years)**					
		**Men (*****n** =* **851)**	**Women (*****n** =* **1,178)**	χ^2^	***P-*** **value**	**18**~**29 (*****n** =* **301)**	**30**~**44 (*****n** =* **837)**	**45**~**59 (*****n** =* **592)**	**60**~**69 (*****n** =* **299)**	χ^2^	***P*** **-value**
Not knowing the salt content of commonly eaten foods (%)	963 (47.46)	394 (46.25)	569 (48.39)	0.796	0.372	146 (48.68)	405 (48.33)	287 (48.48)	125 (41.81)	4.500	0.212
Not knowing how to make food taste good without salt or with less salt (%)	900 (44.36)	375 (44.03)	525 (44.50)	0.050	0.823	124 (40.79)	372 (44.52)	274 (46.28)	130 (43.48)	2.205	0.531
Can't understand the meaning of salt content on food nutrition labels (%)	792 (39.00)	315 (37.12)	477 (40.36)	2.510	0.113	96 (31.58)	304 (36.31)	245 (41.39)	147 (49.16)	23.307	< 0.001
Too few low-salt dishes in restaurants (%)	760 (37.43)	336 (39.46)	424 (35.96)	2.568	0.109	140 (46.38)	350 (41.79)	213 (35.98)	57 (19.06)	61.055	< 0.001
Eat out frequently (%)	559 (27.55)	305 (35.83)	254 (21.57)	50.463	< 0.001	120 (39.80)	268 (32.02)	141 (23.82)	30 (10.03)	81.348	< 0.001
Too few low-salt foods in supermarkets (%)	497 (24.46)	199 (23.30)	298 (25.30)	0.977	0.323	72 (23.68)	250 (29.88)	125 (21.11)	50 (16.72)	26.545	< 0.001

### 3.6. Analysis of the reasons for not taking salt reduction measures

Table 5 revealed that “light taste affects the food texture” and “no harm in eating more salt” were the most and least common justifications for not taking salt reduction measures, respectively (56.71 vs. 5.26%). Men selected “never heard of salt reduction” and “no energy if eating less salt” more frequently than women (all *P* < 0.050). The percentage of women who selected “eat just the right amount of salt” was significantly higher than that of men (*P* = 0.018). A stratified analysis of age groups showed that there were significant differences between age groups with respect to “no energy if eating less salt” and “no harm in eating more salt” (*P* < 0.001). In addition, we noted that there were significant differences in the responses to the statements “never heard of salt reduction” and “light taste affects the texture of food” across age groups (all *P* < 0.050).

**Table 5:** Comparison of reasons for not adopting salt reduction measures among different gender and age groups.

**Reasons for not adopting salt reduction Measures (multiple choices)**	**Total (*n =* 1,349)**	**Gender**				**Age (years)**					
		**Men (*****n** =* **747)**	**Women (*****n** =* **602)**	χ^2^	***P*** **-value**	**18**~**29 (*****n** =* **296)**	**30**~**44 (*****n** =* **541)**	**45**~**59 (*****n** =* **339)**	**60**~**69 (*****n** =* **173)**	χ^2^	***P-*** **value**
Light taste affects the food texture (%)	765 (56.71)	439 (58.77)	326 (54.15)	2.893	0.089	141 (47.64)	314 (58.04)	205 (60.29)	105 (60.69)	13.392	0.004
Eat just the right amount of salt (%)	671 (49.74)	350 (46.85)	321 (53.32)	5.579	0.018	140 (47.30)	277 (51.20)	162 (48.53)	84 (48.55)	1.594	0.661
No need to reduce salt (%)	331 (24.54)	183 (24.50)	148 (24.58)	0.001	0.971	66 (22.30)	138 (25.51)	88 (25.88)	39 (22.54)	1.819	0.611
Never heard of salt reduction (%)	292 (21.65)	188 (25.17)	104 (17.28)	12.241	< 0.001	80 (27.03)	118 (21.81)	56 (16.47)	38 (21.97)	10.326	0.016
No energy if eating less salt (%)	177 (13.12)	118 (15.80)	59 (9.80)	10.513	0.001	19 (6.42)	65 (12.01)	52 (15.29)	41 (23.70)	30.691	< 0.001
No harm in eating more salt (%)	71 (5.26)	47 (6.29)	24 (3.99)	3.552	0.059	9 (3.04)	18 (3.32)	27 (4.99)	17 (9.83)	19.186	< 0.001

## 4. Discussion

According to a survey by Hu et al., in 2019, 60.07% of residents reduced their salt intake, which was an increase from 37.3% among Chinese adults in 2015 (37.3%) (15). Similarly, this study showed that the adoption of salt-reduction strategies by residents was influenced by gender, age, education level, marital status, and history of hypertension.

The findings suggested that women were more motivated to reduce their salt intake, possibly because they were typically in charge of the kitchen and were concerned about their family members' diets, thereby paying more attention to salt-related information (16, 17). The results of previous studies were generally supported by the higher awareness of salt reduction behavior rates among the more educated and older adults (18, 19). The rate of salt reduction behavior was higher in the hypertensive population than in the non-hypertensive population, and this finding was consistent with a study by Arcand et al. (20). This might be because people with hypertension were more likely to have health issues that led them to restrict their daily salt intake or take other measures to minimize their salt intake on the advice of their doctors, resulting in more effective and affordable BP management. Many governments, such as the United States and the United Kingdom, have adopted a series of policies to control salt intake (21). To solve the problem of excess salt, the Chinese government has introduced a series of policies focusing on salt reduction. In the “Healthy China 2030” action plan, the government has set a goal of reducing salt intake by 20% by 2030 (22, 23).

With China's rapid urbanization and lifestyle changes, restaurants have become the second most popular meal option after home-cooked meals; this shift has made eating out a key barrier to population-wide salt reduction (24). There were significant differences in the frequency of dining out among age groups, with younger people possibly preferring to dine out while older people were less likely to do so (18). Additionally, there were also differences in how each gender handled this situation (25). We speculate that men may interact more with others at work than women, making it difficult for them to decline invitations to eat out.

Nitrites contained in pickled products can improve the flavor by inhibiting lipid peroxidation and preventing the food from becoming rancid (26). Reducing salt in food may reduce its taste, making older people hesitant to adopt salt reduction measures (27). Younger people were also more likely to refuse or consume less because they were more likely than older people to be aware that nitrites in pickled foods might have harmful effects on their health (18). Since the majority of product labels only indicate sodium levels, understanding the relationship between sodium and salt might be challenging for people of different ages and cultural backgrounds (28, 29). Therefore, the nutrition label might have some impact on salt reduction across age groups.

Our study has the following strengths: (1) The evidence on salt-reduction-related behaviors was still lacking among residents in the study area, so this study might offer some scientific recommendations; (2) To ensure the representativeness of study subjects, we selected 10 districts and counties that covered a wide area. However, several limitations should be noted in this study: (1) Older people were more likely to suffer from chronic diseases and had received health education related to salt reduction, and their salt intake was relatively lower; (2) The data were self-reported by the respondents and may be subject to some information bias; and (3) We did not ask participants if they had any education about salt reduction, which could have biased the results to some extent.

## 5. Conclusion

In conclusion, the proportion of salt reduction behaviors among the residents of Anhui Province is not high. Women, older individuals, couples, those with bachelor's degrees or higher, and those with high BP have a tendency to consume less salt. By understanding the factors affecting salt reduction measures and the reasons for not adopting them, the government should, to a certain extent, adopt a consistent and feasible strategy to raise awareness of salt reduction for hypertension prevention and control.

## Data availability statement

The raw data supporting the conclusions of this article will be made available by the authors, without undue reservation.

## Ethics statement

The studies involving humans were approved by the Medical Ethics Review Committee of Anhui Provincial Center for Disease Control and Prevention. The studies were conducted in accordance with the local legislation and institutional requirements. The participants provided their written informed consent to participate in this study.

## Author contributions

X-YX and YZ participated in the questionnaire entry and drafted the manuscript. J-QX and Y-JC contributed to the questionnaire survey. H-DW and WX contributed to the statistical analysis. NS contributed to checking the data daily. H-FP and Z-RL conceived of the presented idea, took responsibility for the content of the manuscript, including the conception and design of the study, and final approval of the version to be submitted. All authors contributed to the article and approved the submitted version.
